# PaGenBase: A Pattern Gene Database for the Global and Dynamic Understanding of Gene Function

**DOI:** 10.1371/journal.pone.0080747

**Published:** 2013-12-02

**Authors:** Jian-Bo Pan, Shi-Chang Hu, Dan Shi, Mei-Chun Cai, Yin-Bo Li, Quan Zou, Zhi-Liang Ji

**Affiliations:** 1 State Key Laboratory of Stress Cell Biology, School of Life Sciences, Xiamen University, Xiamen, Fujian, PR China; 2 Department of Chemical Biology, College of Chemistry and Chemical Engineering, The Key Laboratory for Chemical Biology of Fujian Province, Xiamen University, Xiamen, Fujian, PR China; 3 School of Information Science and Technology, Xiamen University, Xiamen, Fujian, PR China; University of Iceland, Iceland

## Abstract

Pattern genes are a group of genes that have a modularized expression behavior under serial physiological conditions. The identification of pattern genes will provide a path toward a global and dynamic understanding of gene functions and their roles in particular biological processes or events, such as development and pathogenesis. In this study, we present PaGenBase, a novel repository for the collection of tissue- and time-specific pattern genes, including specific genes, selective genes, housekeeping genes and repressed genes. The PaGenBase database is now freely accessible at http://bioinf.xmu.edu.cn/PaGenBase/. In the current version (PaGenBase 1.0), the database contains 906,599 pattern genes derived from the literature or from data mining of more than 1,145,277 gene expression profiles in 1,062 distinct samples collected from 11 model organisms. Four statistical parameters were used to quantitatively evaluate the pattern genes. Moreover, three methods (quick search, advanced search and browse) were designed for rapid and customized data retrieval. The potential applications of PaGenBase are also briefly described. In summary, PaGenBase will serve as a resource for the global and dynamic understanding of gene function and will facilitate high-level investigations in a variety of fields, including the study of development, pathogenesis and novel drug discovery.

## Introduction

Gene expression is dependent on physiological conditions, which vary based on cell type, tissue type and developmental stage. The emergence of high-throughput technologies, such as microarrays, tiling arrays and next-generation sequencing (NGS), has enabled thousands of genes to be monitored simultaneously under multiple conditions. From these data, genes with particular expression patterns can be identified. Specific gene patterns may suggest an association with a particular biological event or function. Therefore, the identification of pattern genes holds promise for the dynamic and global investigation of gene function. Moreover, gene pattern studies may help to uncover the molecular mechanisms underlying certain physiological events.

Pattern genes are defined as a group of genes that exhibit modularized expression behavior under serial physiological conditions [1]. Three types of pattern genes are presently attracting significant attention: housekeeping genes, specific/selective genes and repressed genes [1]–[4]. Housekeeping genes, which are expressed ubiquitously across tissues under all physiological conditions and developmental stages, are generally believed to maintain basal cellular functions [2]. Deficiencies or non-synonymous mutations in housekeeping genes will likely lead to disease [5]. Housekeeping genes are typically adopted as molecular controls in qualitative or semi-quantitative measurements of gene expression. Specific/selective genes are genes that are preferentially expressed under one or more conditions [3]. Their enriched expression levels are typically considered markers of the initiation or existence of some biological phenomena, such as development, proliferation, differentiation or pathogenesis [6]–[9]. For example, a systematic analysis demonstrated that many diseases are associated with tissue-specific genes [10], suggesting that these genes may serve as potential biomarkers and targets for disease diagnosis and treatment [11]. In contrast to specific/selective genes, repressed genes are expressed under almost all conditions, except for one or more conditions. The undesired expression of repressed genes may lead to disease, which provides an alternative route for the exploration of the molecular mechanisms underlying pathogenesis [4].

The spatiotemporal pattern of gene expression provides important information for comprehensively understanding the function of genes and also provides useful clues for the systematic investigation of the molecular mechanisms underlying physiology and pathogenesis [12]. Therefore, numerous efforts have been made in recent years to determine gene expression profiles under pre-designed conditions using high-throughput technologies. Accompanying this rapid growth of data, bioinformatics tools have been developed for mining patterns or trends hidden within the data. Many of these studies utilize statistical methods or sophisticated algorithms to ‘align’ or ‘cluster’ gene expression profiles to extract non-trivial patterns, such as correlated expression, differential expression, specific expression and ubiquitous expression [1], [13]–[17]. While other databases have been constructed to collect pattern gene information (such as FlyExpress [18], ZFIN [19], TiSGeD [20], TiGER [21], BioGPS [22] and TissueDistributionDBs [23]), many of these databases rely on a relatively small number of tissue-specific/selective genes or housekeeping genes. Poor or even absent quantitative evaluation limits the ability of these databases to identify pattern genes. We designed four statistical parameters in a previous study that enable the quantitative identification and evaluation of pattern genes [1]. Based on this method, we now introduce a novel subject-specialized database called the Pattern Gene database (PaGenBase) for the integration of pattern genes identified from the literature and from data mining of gene expression profiles.

## Materials and Methods

### The Data

The original microarray, NGS and tiling array datasets along their latest gene annotation files were primarily derived from the public microarray repositories NCBI GEO [13], GNF BioGPS [22] and EBI ArrayExpress [14]. The current version of PaGenBase includes datasets that were selected based on the following criteria: the dataset (i) consists of time or space series samples, (ii) contains at least 5 different samples (excluding duplicates), (iii) contains a relatively large number of genes and (iv) was collected under normal physiological conditions. A total of 143 qualified datasets, which were selected from more than 3,400 datasets, underwent further manual verification. The datasets were cleaned by removing profiles with incomplete or unreliable information. Microarray datasets with Absent/Marginal/Present (A/M/P) calls were normalized by setting the expression value to 0 if the A/M/P call was marked as ‘absent’ or ‘marginal’. The average value was used for duplicate samples. For genes with multiple probesets, the probeset (profile) with the largest standard deviation was selected. After data pre-processing, the datasets were analyzed to identify pattern genes via the method described in our previous work [1], which is also briefly described in the following section. Additional pattern genes were manually collected by keyword searches of the biomedical literature database PubMed. Both the computed results and the literature-extracted information were appropriately formatted before they were uploaded to the database.

### Quantitative Identification of Pattern Genes

To aid in the identification and quantitative evaluation of pattern genes from high-throughput datasets, four statistical parameters, the Specificity Measure (SPM), the Dispersion Measure (DPM), the Contribution Measure (CTM), and the Repression Measure (RPM), were used and are described briefly below. More detailed information on these parameters can be found in our previous work [1].

First, each gene expression profile is transformed into a vector *X*:
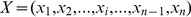
(1)where *n* is the number of samples in the profile. Similarly, a vector *X_i_* can be generated to represent the gene expression in sample (condition) *i*:
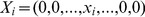
(2)


#### SPM

SPM is a parameter that measures the specificity of a gene's expression in a designated sample. SPM is the cosine value of the intersection angle *θ* between vectors *X_i_* and *X* in high dimensional feature space. It is calculated by the following expression:
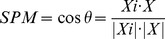
(3)where |*X_i_*| and *|X|* are the length of vectors *X_i_* and *X,* respectively. SPM values range from 0 to 1.0, with values close to 1.0 indicating a major contribution to gene expression in a designated sample (vector *X_i_*) relative to all samples (vector *X*). A gene expression profile (*X*) can be converted to a corresponding SPM profile (*X_SPM_*):
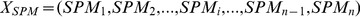
(4)


#### DPM

DPM is the standard deviation in unitary form based on the transformed SPM profile. DPM enables the comparison of variances between two profiles regardless of their absolute expression values. It is calculated by the following expression:
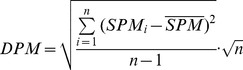
(5)where 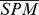 is the mean value of *SPM*s in a gene expression profile. DPM values close to 0 suggest nearly equal gene expression levels across all samples.

#### CTM

CTM, which is complementary to SPM, is a parameter that measures the enrichment of gene expression levels in several samples. It is calculated by the following expression:
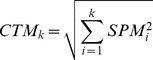
(6)where *k* is the number of selected samples. Typically, *k* ranges from 2 to 6. CTM values range from 0 to 1.0, with values close to 1.0 indicating enriched expression levels in select samples.

#### RPM

RPM is a parameter designed to evaluate the significance of repressed gene expression levels. First, the gene expression profile is divided into two groups: (i) the user-defined *k* samples (typically ranging from 1 to 6) containing the expected repressed expression level (normally, *k* is taken from the lowest expressions in the profile) and (ii) the remaining samples. Then, RPM is calculated by the following expression:
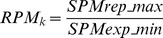
(7)where *SPMrep_max* is the maximum SPM value in the putative repressed expression group and *SPMexp_min* is the minimum SPM value in the expression group. RPM values range from 0 to 1.0, with values close to 0 indicating a significantly lower expression of a given gene in the user-defined *k* samples compared to the other samples.

Pattern genes can be defined and evaluated using the four statistical parameters defined above (SPM, DPM, CTM and RPM) alone or in combination. Examples of how these parameters can be applied are provided for reference. A housekeeping gene is ubiquitously and nearly equally expressed in all selective samples (e.g., DPM<0.3). A specific gene is specifically expressed in one sample (e.g., SPM>0.9). A selective gene has an enriched expression in limited *k* samples (e.g., 2≤*k*≤6, SPM*_i (1 to k)_*>0.3, and CTM*_k_*>0.9). In contrast, a repressed gene has no or very low expression in limited *k* samples (e.g., 1≤*k*≤6, SPM*_i (1 to k)_*<0.1, and RPM*_k_*<0.2).

In addition to the SPM method, the conventional proportion (*r_i_*) method is also used in PaGenBase to measure the fractional gene expression in a defined sample *i* against the entire gene expression profile:
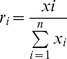
(8)where *n* is the number of samples in a profile. Accordingly, an additional parameter called *S*UM is used to measure the enrichment of gene expression in several samples (gene selectivity) by calculating the sum of fractional expression levels in selective samples. SUM is calculated by the following expression:
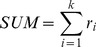
(9)


Compared to the proportion method *r_i_*/SUM, SPM/CTM is more sensitive for identifying specific/selective genes.

## Results

### Retrieval of Pattern Genes from PaGenBase

The PaGenBase database can be accessed at http://bioinf.xmu.edu.cn/PaGenBase/. The database runs on RDBMS Oracle *10 g* and the *Red Hat Linux release 9* operating system. User-friendly interfaces and search engines were implemented using JSP technology. The three data retrieval methods that were developed for accessing PaGenBase are briefly described below.

#### The quick search and advanced search methods

**Figure 1:**
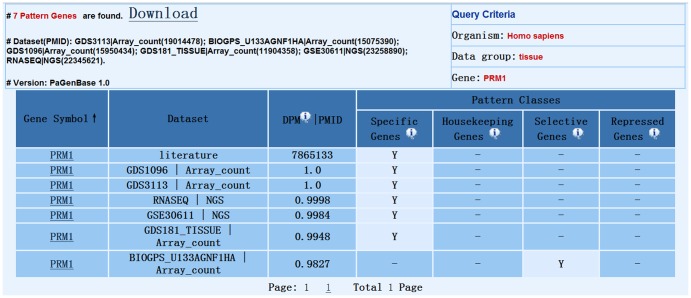
The Pattern Gene Table Page.

**Figure 2:**
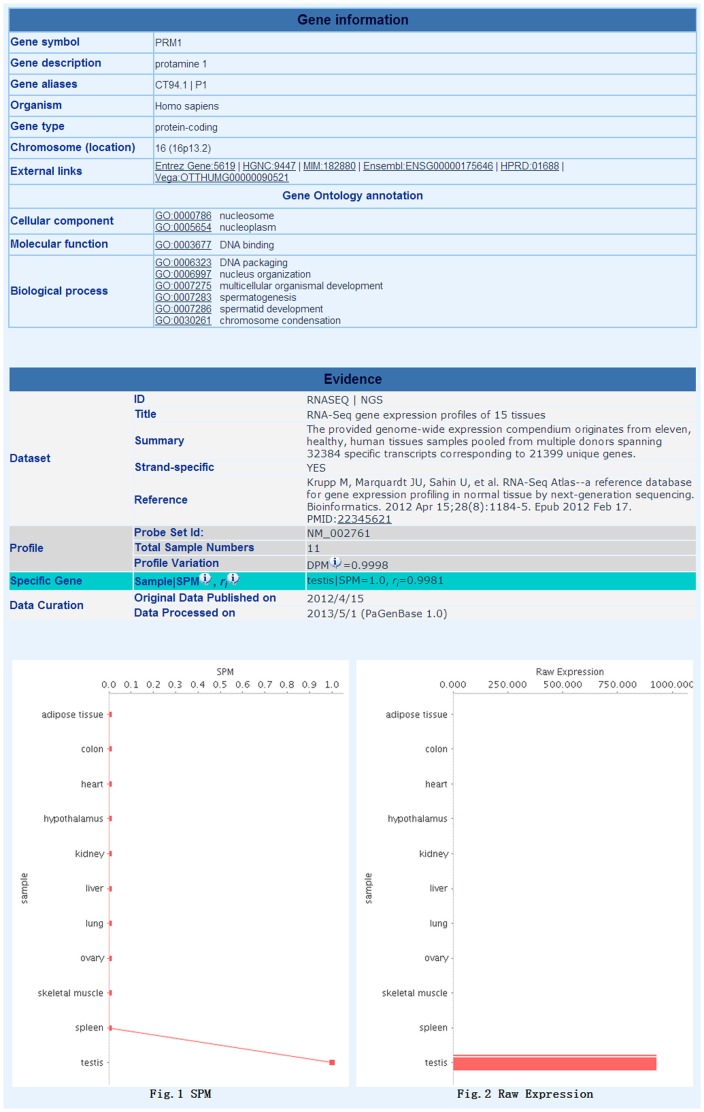
The Detailed Information Page.

For the rapid retrieval of pattern genes, PaGenBase provides a quick search method on the homepage. Via the quick search form, the user can retrieve pattern genes in the selected organism and data group by providing a keyword. The selection of an organism and a subsequent data group is required for a database search, after which a complete or partial keyword for a gene or sample is requested to initiate the search. Wildcard characters such as ‘* & ?’ are not supported. Due to the lack of available of data, some organisms currently have only one or two datasets and data groups. The quick search method will automatically adopt the default query criteria for pattern genes: SPM>0.9 for specific genes; DPM<0.3 for housekeeping genes; sample number = 2∼6, SPM_sample_>0.3 and CTM>0.9 for selective genes; and sample number = 1∼6, SPM_sample_<0.1 and RPM<0.2 for repressed genes. Genes or samples that meet the query criteria will be presented in ascending alphabetical order. However, if no keyword was input, the database will list all pattern genes or samples in alphabetical order. The number of pattern genes or samples is provided in the left corner of the page. Clicking on a gene symbol or sample name will redirect the user to the Pattern Gene Table Page (**Figure 1**), where the selected pattern gene(s) will be listed along with information on the dataset, DPM value, the PubMed ID (PMID) (if the pattern gene was derived from the literature) and the pattern type. This table can be downloaded via the ‘Download’ hyperlink in the left corner of the page. Clicking on the gene symbol will lead to the Detailed Information Page (**Figure 2**), where two sections of information are provided: (1) the ‘Gene information’ section, which provides basic information on the gene, such as the gene symbol, chromosomal location and gene ontology as well as external links to some well-known databases, such as the NCBI Entrez Gene database [24] and Ensembl [25], when available; and (2) the ‘Evidence’ section, which provides experimental evidence on pattern genes, including the dataset description, profile information, pattern genes, and their quantitative measure. To enable user tracking, the dataset processing history and the data curation record is provided in the ‘Evidence’ section, which includes the original data publication date and the data processing date. The visualization of gene profiles (both raw data and SPM transformed data) is also provided. Literature evidence is also presented in this section when available, which includes the experimental method used and the associated PMID.

**Figure 3:**
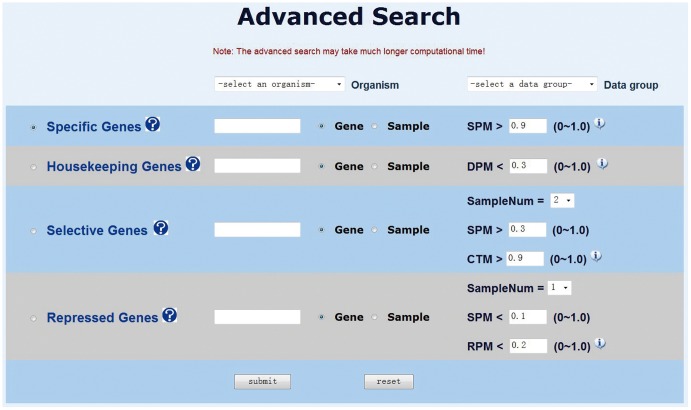
The Advanced Search Method.

In addition, PaGenBase offers an advanced search method for the customized retrieval of pattern genes (**Figure 3**). The advanced search method works in almost the same manner as the quick search method except that the user is allowed to customize the query criteria by pattern gene types rather than default values. It should be noted that a customized query criteria will sometimes require more computational time to retrieve results.

#### The browse method

**Figure 4:**
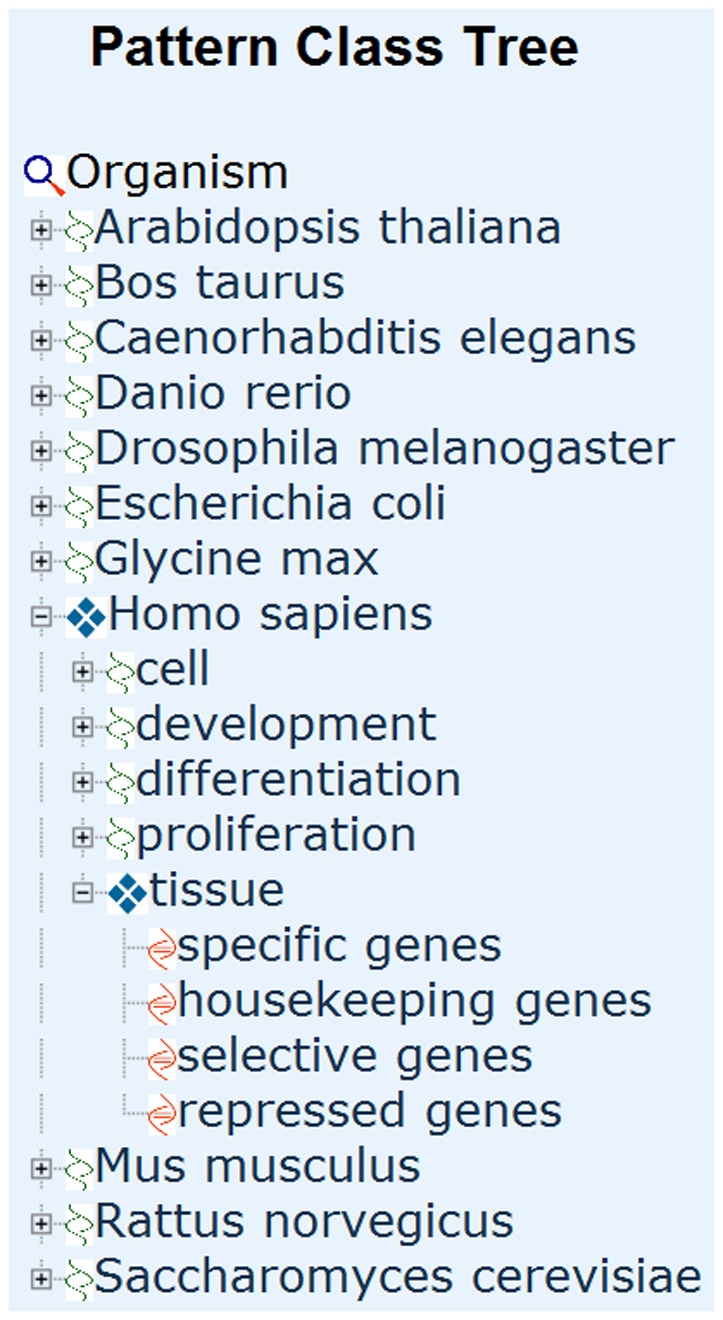
The Browse Method.

Alternatively, PaGenBase offers the browse method for the direct retrieval of information from the database (**Figure 4**). All pattern genes are non-redundantly arranged into a tree. The tree is expandable up to a maximum of three levels. The first level includes 11 organisms, such as *Homo sapiens*. The second level includes one or more data groups, such as tissue and development. The third level includes four pattern classes: specific genes, selective genes, housekeeping genes and repressed genes. Selecting a pattern class will redirect the user to the Pattern Gene Table Page. In this table, all pattern genes of the selected pattern class are listed. Clicking on the gene symbol will lead to the Detailed Information Page. Similar to the quick search method, the browse method adopts the default query criteria for all four pattern gene classes.

#### Global understanding of gene expression patterns

In PaGenBase, the majority of pattern genes have been identified by comparing their expression patterns under serial conditions, such as in different tissues or developmental stages. All differential expression patterns were considered as a whole. However, only the most obvious changes are indicated, which will save the user the time required to manually compare gene expression changes between conditions or genes. The user can then rapidly focus on the most important gene changes across the conditions. Moreover, PaGenBase offers a platform for the global understanding of a gene by integrating information from multiple high-throughput datasets. For example, the protamine 1 (PRM1) gene encodes a small, arginine-rich, nuclear protein that replaces histones in the chromatin of sperm during the haploid phase of spermatogenesis [26]. This gene was found to be specifically expressed in the testis (SPM_testis_ = 1.0) based on microarray datasets GDS3113 [11] and RNASEQ (**Figure 2**) [27]. The specific expression of PRM1 can be further specified by analyzing the dataset BioGPS U133A GNF1HA (GDS596), where the gene was actually determined to be highly expressed in the sub-tissues of the testis interstitium, testis seminiferous tubules, and testis (SPM_Testis Interstitium_ = 0.66, SPM_Testis Seminiferous Tubule_ = 0.47, SPM_Testis_ = 0.59, and CTM = 1.0) [28]. In human cell lines (dataset BioGPS U133A GNF1HB), PRM1 is selectively expressed in testis leydig cells and testis germ cells (SPM_Testis Leydig Cell_ = 0.78, SPM_Testis Germ Cell_ = 0.62, and CTM = 1.0) [28]. These tissue/cell distribution data support the previous findings that PRM1 is likely associated with postmeiotic germ cells [29]. Furthermore, through mining the GDS605 dataset, which was collected under a mouse spermatogenesis and testis development time course, PRM1 was found to be selectively expressed during testis development at days 30, 35, and 56 (SPM_day30_ = 0.50, SPM_day35_ = 0.52, SPM_day65_ = 0.69, and CTM = 1.0), corresponding to the spermatogenesis stages of spermatids and spermatozoa [30]. As a direct consequence of aggregating all these data, it can be proposed that PRM1 plays a role in the postmeiotic stage of spermatogenesis.

### The Statistics of PaGenBase

Currently, PaGenBase release 1.0 contains 143 distinct datasets, 1,145,277 valid gene expression profiles and 119,538 annotated genes collected from the following 11 model organisms: *Arabidopsis thaliana*, *Bos taurus*, *Caenorhabditis elegans*, *Danio rerio*, *Drosophila melanogaster*, *Escherichia coli*, *Glycine max*, *Homo sapiens*, *Mus musculus*, *Rattus norvegicus* and *Saccharomyces cerevisiae*. Based on the experimental conditions used, each dataset was pre-assigned into one of nine data groups, including cell line, cell cycle, condition, development, differentiation, proliferation, strain, tissue type and others. A total of 906,599 pattern genes (and 111,897 distinct genes) were identified in 1,062 distinct tissue and time specific conditions, which includes 145,270 specific genes, 468,207 housekeeping genes, 156,974 selective genes and 136,148 repressed genes. An additional 323 pattern genes were derived from the literature. The detailed statistics of the database are provided in **Table 1** and on the ‘STATISTICS’ page.

**Table 1:** Statistics of the PaGenBase database.

Organisms	Genes	Samples	Datasets	Profiles	Specific Genes*	Housekeeping Genes*	Selective Genes*	Repressed Genes*	Pattern Genes*
*Arabidopsis thaliana*	14,312	6	1	14,312	694 (694)	9,614 (9,614)	998 (998)	1,052 (1,052)	12,358 (11,996)
*Bos taurus*	1,092	17	2	1,123	14 (14)	913 (893)	2 (2)	10 (10)	939 (918)
*Caenorhabditis elegans*	15,861	7	1	15,861	784 (784)	6,512 (6,512)	946 (946)	4,620 (4,620)	12,862 (12,581)
*Danio rerio*	9,984	11	2	19,962	97 (97)	13,820 (9,955)	634 (634)	266 (266)	14,817 (9,964)
*Drosophila melanogaster*	12,643	54	9	75,307	11,318 (7,724)	25,060 (10,673)	9,271 (6,332)	10,786 (6,939)	56,435 (12,554)
*Escherichia coli*	4,117	22	6	23,671	276 (228)	15,529 (4,023)	573 (455)	2,101 (1500)	18,479 (4070)
*Glycine max*	2,102	26	3	5,981	1,599 (989)	378 (239)	1,455 (961)	909 (624)	4,341 (1,792)
*Homo sapiens*	26,014	305	26	278,754	36,351 (16,176)	113,719 (21,490)	34,186 (15,276)	25,987 (14,165)	210,243 (25,162)
*Mus musculus*	23,453	425	78	643,694	91,663 (18,503)	249,286 (22,872)	104,323 (19,169)	84,305 (16,730)	529,577 (23,214)
*Rattus norvegicus*	4,020	43	3	9,967	1,970 (1,546)	2,003 (1,230)	2,402 (1,730)	1,594 (1,344)	7,969 (3,790)
*Saccharomyces cerevisiae*	5,940	146	12	56,645	504 (455)	31,373 (5,834)	2,184 (1,495)	4,518 (3,326)	38,579 (5,856)
Total	119,538	1,062	143	1,145,277	145,270 (47,210)	468,207 (93,335)	156,974 (47,998)	136,148 (50,576)	906,599 (111,897)

* Numbers in parentheses indicate the number of non-redundant genes.

### Database Comparison and Updating

PaGenBase is a novel database that integrates pattern genes quantitatively identified under various tissue and time specific conditions. After surveying the internet, we determined that no other public database currently provides a similar service. However, some existing databases contain useful information on pattern genes. For example, TiGER [21], FlyExpress [18], the Human Protein Atlas [31] and ZFIN [19] provide comprehensive genome/proteome, transcriptome, *in situ* hybridization and other information for generating a systematic understanding of a model organism. While some bioinformatics tools exist for the visualization and comparison of gene expression patterns across tissues and developmental stages, none of these tools provide quantitative identification of modular expression genes. TiSGeD, a database developed by our research group two years ago, provides information on tissue-specific genes. This database, which served as the prototype for PaGenBase, contains a smaller number of data types (only tissue-specific genes) and contains only 7 microarray datasets. TiGER is another database containing tissue-selective genes that uses a fractional method to evaluate genes that are preferentially expressed in a designated tissue. A similar approach was adopted by TissueDistributionDBs [23]. Compared to previously developed pattern gene-related databases, PaGenBase has several advantages: (i) in addition to specific/selective genes and housekeeping genes, PaGenBase also provides information on repressed genes; (ii) PaGenBase collects pattern genes identified on a large scale from gene expression profiles under serial experimental conditions in not only different tissues but also under various conditions, such as different developmental stages; (iii) unlike previous databases, such as TiGER, which use limited EST and microarray data for the assessment of tissue-specific expression, the current release of PaGenBase contains 143 quality microarray, NGS and tiling array datasets from the literature; and (iv) PaGenBase is currently the only database that provides statistical measurements for all pattern gene classes. Moreover, some general repositories, such as GEO [13], ArrayExpress [14], and BioGPS [22], contain a large number of expression profiles and serve as a major data sources for PaGenBase. Although these repositories also implement powerful statistical tools, none provide direct information on pattern genes like PaGenBase does.

PaGenBase is scheduled to be updated continually and regularly. Importantly, more quality datasets, especially new high-throughput datasets, will be incorporated into the database as they are available. The planned expansion of the database will not only incorporate more datasets from various data sources but will also cover more experimental conditions, such as pathogenic samples. In the current version of the database, a discrepancy in pattern gene assignments exists. Information about the discrepancy can be found on the Pattern Gene Table Page, where all expression patterns for a gene are listed. The discrepancy can largely be attributed to the inclusion of heterogeneous datasets that differ in important aspects of sample preparation, experimental design, and analysis platform. For example, PRM1 was identified as a testis-specific gene in several datasets, including GDS3113 [11] and RNASEQ [27] (**Figure 1** **&** **2**); however, PRM1 was also identified as a selective gene in testis interstitium and testis seminiferous tubules based on the BioGPS U133A GNF1HA (GDS596) dataset [28] (**Figure 1**). In this case, key differences in sample preparation contributed to the PRM1 discrepancy. To solve the discrepancy problem, a scoring system is planned for the next major update to PaGenBase, which will enable better integration of results derived from heterogeneous datasets. Gene expression patterns will be suggested according to the consistency of different assignments. This improvement will provide more reliable information by reducing the possible false assignment of pattern genes due to imperfect experimental design or erratic expression. Moreover, understanding how genes interact with other genes in different conditions carries great promise. Better user interfaces and analytical tools that will enable better gene comparison are also planned for a future release. For instance, similarity and correlation analyses between two gene expression profiles will be implemented [32].

## Discussion

Genes are not static information carriers but instead exhibit varied expression levels in response to internal or external environmental changes, the process by which genes exert control over molecular function. For a better understanding of gene function, it has been suggested that gene expression changes should be monitored under serial tissue and time specific conditions. Differential analyses can be efficiently applied to detect significantly changed genes compared to a control condition. However, differential analysis is inadequate to address some fundamental questions. For example, is gene expression status consistent with a certain condition or is it a common phenomenon? How does a gene perform during the course of a condition change, such as in different developmental stages? Is there any connection between variably expressed genes? What could the regulatory mechanism underlying the different physiological conditions be? Therefore, a global and dynamic investigative tool built from a large number of gene expression profiles is desired. PaGenBase is just one resource that is capable of providing this type of information.

### Selection of Reference Genes

Housekeeping genes, such as peptidyl-prolyl cis-transisomerase A (PPIA), glyceraldehyde-3-phosphate dehydrogenase (GAPDH) and beta-actin (ACTB), are widely employed as molecular controls for qualitatively or semi-quantitatively measuring gene expression. This approach is based on the assumption that housekeeping genes are expressed at a constant level under different physiological conditions. For a relatively long time, few doubts were raised about whether the expression of these genes is actually constant. In a recent genome-wide study, it was determined that many housekeeping genes, such as PPIA, GAPDH and ACTB, are not consistently expressed across tissues under a normal physiological state or experimental conditions [33]. Our analysis on dataset GDS3113 supports the finding that PPIA, ACTB, and GAPDH are not consistently expressed in human tissues (their DPM values were 0.31, 0.35 and 0.50, respectively) [11]. In particular, GAPDH is selectively expressed in skeletal muscle (SPM_skeletal muscle_ = 0.47). This result is consistent with the finding of Barber's group, who measured GAPDH mRNA expressions in 72 human tissues via quantitative real-time RT-PCR [34]. They found a 15-fold difference in GAPDH mRNA copy number between the highest and lowest expressing tissue types, which were skeletal muscle and breast, respectively. Therefore, it may not be appropriate to use traditional housekeeping genes as a quantitative reference for comparing gene expression profiles across tissues. The question then becomes how to choose a good reference gene. First, a reference gene should be ubiquitously expressed under all studied conditions. Second, it should have relatively high expression levels so that it can be easily and stably detected [35]. Low gene expression levels are often obscured by background noise or erratic signals. Third, its expression should be insensitive to condition changes. The consistent expression of a gene guarantees it as a good reference control without bias among different experimental conditions. The consistency of a gene's expression is usually measured by algorithms, such as geometric mean, standard deviation or linear regression [36], [37]. In this study, the transformed standard deviation method, DPM, was adopted, which is useful regardless of the absolute gene expression level. A typical example of a housekeeping gene is the golgin A1 gene (GOLGA1), which was found to be consistently expressed in 26 human tissues (Dataset GDS3113: DPM_GOLGA1_ = 0.28, Raw average value_RPL37_ = 6,675) in this study. This result is supported by Soyoun's work on the identification of universal housekeeping genes from human microarray (HG-U133; Affymetrix) data using several approaches, including the geometric mean [37]. It should be noted that determining a good reference housekeeping gene is subject to experimental conditions. For example, GAPDH may not be a good reference across multiple human tissues because it is selectively expressed in skeletal muscle. However, GAPDH expression is nearly unchanged in some cell types as well as during distinct phases of the menstrual cycle, which could make it a good reference gene in some studies.

### Identification of Biomarkers

Gene biomarkers are routinely used in many areas of modern biomedical research and in a variety of clinical applications. It is generally acknowledged that a typical gene biomarker is an indicator of a specific biological state [38]. For example, PRM1 is a potential biomarker for spermatogenesis and the diagnosis of male infertility [39]. A recent study successfully used our specificity measure method (SPM) to aid in the identification of pancreatic cancer biomarkers [40]. Therefore, PaGenBase can serve as a good resource for the identification of candidate gene biomarkers because it can collect thousands of specific genes under a variety of physiological conditions.

## Conclusions

In this study, we introduced a novel database that contains pattern genes identified from serial transcriptomes under multiple physiological conditions. This database is the most comprehensive public repository presently available for identifying modularized gene expression patterns. PaGenBase will serve as a useful resource for global and dynamic understanding of gene behavior under a variety of tissue and time specific conditions, especially the developmental stages of tissues and cells. Moreover, the database can be used to build connections between gene and tissue function, organ development, cell proliferation and differentiation. Finally, additional investigations into pattern genes and their protein products may suggest potential applications for disease diagnosis and the discovery of novel drug targets.
